# Effect of technique and transfer board use on the performance of wheelchair transfers

**DOI:** 10.1049/htl.2017.0075

**Published:** 2018-03-05

**Authors:** Giulia Barbareschi, Tsu-Jui Cheng, Catherine Holloway

**Affiliations:** 1 University College London Interaction Centre, University College London, London WC1E 6EA, UK; 2 Centre for Health Sciences Research, University of Salford, Salford M6 6PU, UK

**Keywords:** handicapped aids, biomechanics, injuries, medical signal processing, wheelchair transfers, wheelchair seat, falling, upper limb injuries, spinal cord injury, sitting, standing, transfer board, ground reaction forces, transfer quality, transfer performance, transfer assessment instrument, TAI, mean reaction forces

## Abstract

Transferring to and from the wheelchair seat is a necessary skill for many wheelchair users who wish to be independent of their everyday life. The performance of wheelchair transfers has been associated with the risk of falling and developing upper limb injuries. Both present a risk to the independence of the individual. Previous studies on wheelchair transfers have focused mainly on the analysis of sitting transfers performed by individuals with spinal cord injury, which only represent a small portion of the wider wheelchair users’ population. The purpose of this study is to investigate the effect of different transferring techniques (sitting, standing) and transfer board use on the ground reaction forces under the hands during transfer performance and transfer quality measured using the transfer assessment instrument (TAI). Sitting transfers displayed generally higher peak and mean reaction forces underneath both leading and trailing hands compared with the other techniques, but the difference was only significant between sitting and standing transfers. Standing transfers had significantly lower TAI scores compared with sitting transfer, potentially indicating a decreased level of safety associated with their performance. Transfer boards were only partially effective in reducing the weight born by the upper limbs and they caused only a minor reduction in the overall TAI score in comparison to sitting transfers.

## 1 Introduction

Performing a transfer to and from the wheelchair seat is a necessary skill for wheelchair users who want to complete independently many activities of daily living [1]. The number of wheelchair transfers performed daily by wheelchair users varies greatly, with some studies reporting an average number as low as eight transfers per day [2], while others estimate numbers which are closer to 20 transfers per day [3]. Although it might seem surprising at first, this large variation should be expected. Wheelchair users are a very diverse population that encompass individuals of different sex, age and medical conditions but, more importantly, different functional abilities and lifestyles [4]. Most studies on wheelchair transfers focus mainly on the analysis of sitting pivot transfers performed by individuals with spinal cord injury (SCI) [5–8]. Although individuals with SCI represent a significant group within the wheelchair users’ population, they are not representative of the full population. Sitting pivot transfers are routinely performed by wheelchair users without an SCI and inclusion criteria for studies on wheelchair transfers should be based on functional ability rather than medical condition in order to produce more generalisable results [9]. Additionally, many wheelchair users might be able to reach a standing position and perform a standing pivot transfer [10], while others might use a transfer board in one or more circumstances in order to facilitate transfer performance [11]. Surprisingly, although the use of transfer boards is recommended in order to reduce reaction forces during independent transfers, the effect of using a transfer board on these transfers has never been measured [12]. The few studies that evaluated the effectiveness of transfer boards on reducing forces during wheelchair transfers were carried out on assisted transfers of dependent patients performed by health operators [13, 14].

The performance of wheelchair transfers has been shown to be related to two different risk factors for wheelchair users. Firstly, sitting wheelchair transfers have been linked to pain and injury in the upper limbs [15, 16]. A study from [17] showed how repeated wheelchair transfers cause acute damage to the shoulder tendons that might accumulate over time and cause the onset of overuse injuries. Similar results were described in [18] where ultrasound examination after repeated transfers revealed increases cross-sectional area (CSA) of the median nerve which could, overtime, lead to development of carpal tunnel syndrome. Overuse upper limb injuries among wheelchair users are believed to be related to the high forces generated during activities such as propulsion and transfers [19]. Due to the experiment design, the researchers in [17] are unable to establish any connection between the reaction forces generated during transfers and the ultrasound findings. On the other hand, this relationship appears quite clear in [18] as subjects with increased body mass, who are likely to exhibit greater reaction forces, had increased CSA after transfers. Additionally, results showed how individuals who performed transfers with better technique had a decreased swelling ratio of the median nerve after the experiment. A recent study from [8] seems to confirm the importance of correct technique for subjects performing sitting pivot transfers as it found that subjects who performed better transfers were less likely to complain of shoulder pain and present signs of shoulder pathology during ultrasound examination.

Secondly, wheelchair transfers have been identified as one of the main wheelchair activities that can lead to falls, potentially causing traumatic injuries to the individual and decrease their level of confidence [20–24]. Although the risk of developing upper limb injuries might be more relevant to people who perform sitting wheelchair transfers, the risk of falling while performing a transfer is equally relevant for individuals performing standing transfers. To our knowledge, no clinical scale has been developed to evaluate the risk of falling during the performance of standing and sitting transfers performed independently or with the aid of a transfer board. However, researchers in [25] have developed and refined [26] a clinical tool called transfer assessment instrument (TAI) that can be used to evaluate the performance of independent and assisted wheelchair transfers performed with a standing or sitting technique with or without the aid of a transfer board. The TAI provides a valid and reliable tool to assess aspects of transfer performance which includes the strategy for conservation of the upper limb and the safety of the transfer.

This study aims to evaluate the effect of different transferring techniques (sitting, standing) and transfer board use on the reaction forces under the hands as measured during transfer performance and TAI score. These measures were chosen as they represent an indicator of the risk factors for falling and upper limb injury which are normally associated with wheelchair transfers.

## 2 Methods

### 2.1 Subjects

The study received ethics approval from the University College London Ethics Committee (Study number 4271/002). Participants were recruited from a laboratory database, national and local charities. After reading and providing informed consent, six manual wheelchair users and one power wheelchair user (six males, one female) participated in the study. Inclusion criteria were: aged between 18 and 85 years, use of a manual or powered wheelchair as a primary mean of mobility, ability to perform independent transfer (sitting or standing) with or without the use of a transfer board and no UE pain or injury that would affect their ability to transfer.

### 2.2 Experimental protocol

The majority (six) of our participants were manual wheelchair users. All subjects were asked to perform a transfer from their wheelchair to a transfer bench, then transfer back into their own wheelchair, twice. We attempted to match the height of the bench onto which the wheelchair user would transfer to that of a rigid-frame wheelchair in a standard set-up with pressure-relief cushion. The height of the transfer bench was 55 cm. Transfers were level for nearly all participants and only one subject (Subject 2) had to perform a transfer with a height gap >3 cm (7 cm). Subjects were instructed to freely approach the bench and position the wheelchair at a distance and angle that they were comfortable with. They were also asked after each transfer if they wished to reposition the wheelchair before performing a new SPT, including switching side in order to maintain a consistent leading and trailing arm. After a familiarisation period, two transfers were recorded: wheelchair to bench, bench to wheelchair. If they were familiar with the use of a transfer board, we asked participants who performed sitting transfers to complete the third and fourth transfer using a standard wooden curved transfer board (length 72 cm and width 25 cm). If the subject agreed to use the transfer board, additional practice time was granted before the recording of the following two transfers.

### 2.3 Data analysis

Video recording of transfers was collected using two USB Logitech C930e Webcam (Logitech Europe S.A., CH) positioned at different angles in order to capture the transfers. Videos were used to assess the quality of the transfers using the TAI. The evaluation was carried out independently by two trained physiotherapist using Part 1 of the TAI version 3.0 [26] (Appendix 1). This strategy was chosen as each transfer is scored individually in Part 1 of the TAI, while Part 2 evaluates the summary of the performance of four transfers. Additionally, the final score of the TAI, that includes Parts 1 and 2, has been shown to be highly correlated to the score for Part 1 [27]. Both physiotherapists completed the evaluation separately at first, any disagreement over item score was then resolved through consensus meetings. Items 4, 5 and 15 of the TAI were removed from the evaluation as they were not applicable to any of the recruited participants.

**Fig. 1:**
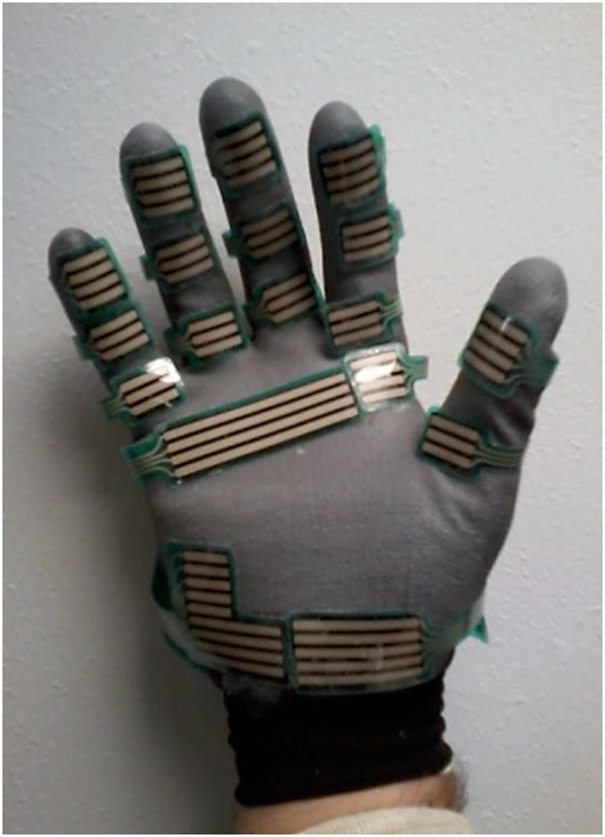
Position of the sensors on the force sensing glove

**Fig. 2:**
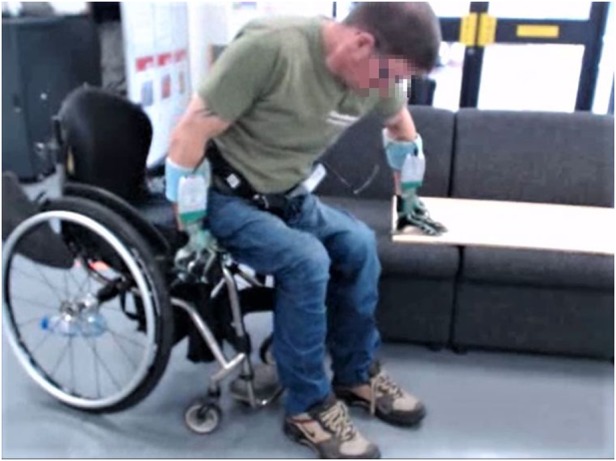
Set-up of the experiment

When quantifying vertical reaction forces under both leading and trailing hands, we wished to avoid constraining the transfer technique in any way. For this reason, instead of measuring reaction forces using force platforms with fixed placements, we opted for asking all subjects to wear a pair of polyurethane gloves that had attached the Tekscan Grip System (Tekscan South Boston, MA, USA) (Fig. 1). To guarantee accurate force measurement, we placed a wooden board of 0.5 cm thickness on the transfer bench. The sampling frequency for the Tekscan system was 25 Hz. The complete set up for the experiment is shown in Fig. 2.

Analysis of the Tekscan data was completed using a custom Matlab script (Matlab 2015b, Mathworks, Inc., Natwick, MA, USA). Reaction forces were normalised as a percentage of body weight as previously recommended by [5]. The gloves were calibrated according to the manufacturer instructions. For each transfer, the peak and mean forces above a threshold of 20 N were calculated for both leading and trailing hand. Force values <20 N were eliminated from the calculation of transfer mean. This threshold was arrived at after consulting the video and concluding that forces under 20 N were often due to baseline noise of the sensors or to contact between the hands and other surfaces (hands resting on thighs).

### 2.4 Statistical analysis

The means and standard deviations of demographic characteristics were computed. Mean TAI score, peak and mean forces for both hands were calculated for each transfer. These were then averaged across individuals for each transfer technique. A one-way analysis of variance was used to assess the effect of technique and transfer board use on mean TAI score, peak and mean forces under leading and trailing hands. Tukey tests was used for post hoc analysis when significant differences were found. The Pearson correlation coefficient was calculated between the mean TAI score and both peak and mean forces for leading and trailing hands. Finally, we used a paired *t*-test to evaluate the effect of transfer board use on transfers performed by the same individual. The level of significance for all tests was set at 0.05. The statistical analysis was performed using SPSS 24 statistical software (SPSS Inc., Chicago, IL, USA).

## 3 Results

### 3.1 Subjects

Demographic characteristics of participants are provided in Table 1

**Table 1:** Demographic characteristics of participants

Subject	Medical condition	Transferring technique	Height, cm	Weight, kg	Gender	Age	Years of use	Type of wheelchair
1	double BKA	standing	177.8	84.5	M	77	9	manual
2	multiple sclerosis	standing	188.9	95.4	M	58	5	electric
3	SCI T9	sitting	180.3	63.3	M	56	32	manual
4	SCI T12	sitting	195.6	92.7	M	28	7	manual
5	EDS-Marfans	sitting	182.9	75	M	26	1	manual
6	SCI T4	sitting	180.2	58.8	M	39	22	manual
7	endometriosis stage IV	sitting	170.2	70.4	F	25	3	manual
Mean			182.3	77.2		44.1	11.3	
SD			8.1	14.2		20	11.4	

Only subjects 3 and 4 could perform the last two transfers with the aid of a transfer board as they had received appropriate training for it during their rehabilitation and were familiar with its use.

### 3.2 Effect of technique and transfer board

Means and standard deviation for TAI score, peak and mean reaction forces for each group are displayed in Table 2 (forces value is reported in BW%).

**Table 2:** Descriptive statistics of TAI score and forces for different techniques

Technique	Value	Minimum, %BW	Maximum, %BW	Mean, %BW	Standard deviation, %BW
standing	peak leading	11.7	18.0	14.8	4.5
	peak trailing	14.3	22.2	18.2	5.6
	mean leading	4.6	6.0	5.3	1.0
	mean trailing	8.2	8.8	8.5	0.4
	TAI score	4.9	4.9	4.9	0.3
sitting	peak leading	24.4	35.9	32.4	4.7
	peak trailing	28.3	41.3	36.8	5.2
	mean leading	9.7	14.4	11.4	2.2
	mean trailing	11.1	16.5	13.8	2.0
	TAI score	6.1	8.2	7.2	0.8
transfer board	peak leading	29.3	30.7	30.0	1.0
	peak trailing	35.5	35.7	35.6	0.1
	mean leading	11.4	12.2	11.8	0.6
	mean trailing	11.7	12.3	12.0	0.4
	TAI score	4.6	6.3	5.5	1.2

As expected, sitting transfers displayed generally higher peak and mean reaction forces under both hands. Standing transfers significantly reduced peak reaction forces under the leading and trailing hands. This was further confirmed by post hoc analysis showing, a consistent, significant decrease in force when completing a standing transfer compared with the other techniques (highest *p*-value 0.03). Reaction forces were not significant between transfers performed with a sitting technique or with a transfer board (lowest *p*-value 0.4). Interestingly, we found that the TAI score was negatively affected by the performance of transfers with a standing technique and with a transfer board. However, this difference was only significant between sitting and standing transfers (*p*-value 0.04). Paired *t*-test showed a similar trend. Mean differences between peak and mean reaction forces under the leading and trailing hands and between sitting and transfer board transfers were, respectively, 3.3 ± 3.0 (peak leading), 3.3 ± 2.0 (peak trailing), 2.0 ± 0.7 (mean leading), 0.1 ± 1.8 (mean trailing), while the mean difference for TAI score was −1.7 ± 0.6. However, none of these differences was found to be significant.

### 3.3 Relationship between TAI score and reaction forces

**Fig. 3:**
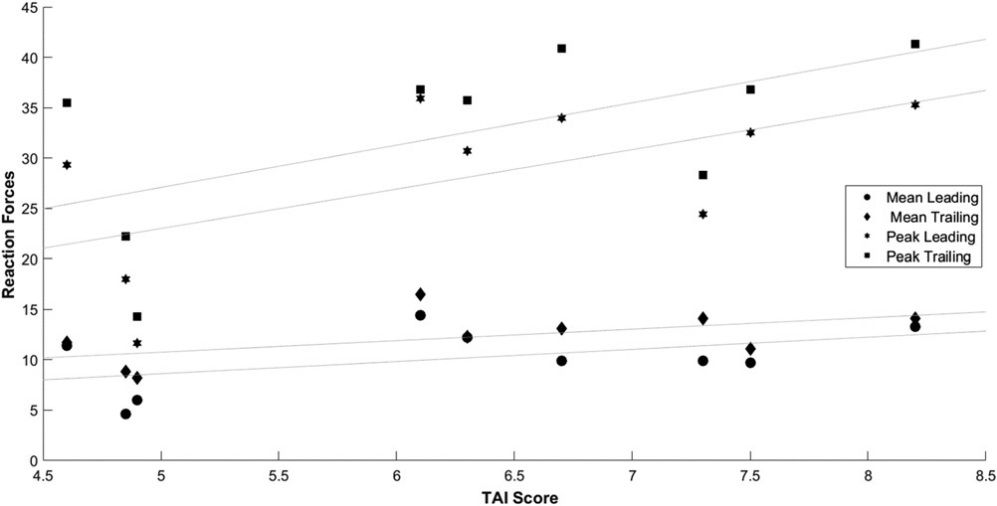
Scatter plot showing the relationship between reaction forces and TAI score across all groups

When analysed across all techniques, we found no significant correlation between reaction forces and TAI score (lowest *p*-value 0.09), we were initially surprised to notice that there was a positive trend between higher TAI scores and greater reaction forces underneath both leading and trailing hands (Fig. 3).

However, when sitting transfers are examined separately, they exhibit an inverse trend where negative correlations are observed between the TAI score, peak leading hand reaction forces (−0.2), mean leading hand reaction forces (−0.2) and mean trailing hand reaction forces (−0.5). Peak trailing hand reaction forces showed a mildly positive correlation (0.1) with the TAI score. None of the above correlations between the TAI score and reaction forces for sitting transfers was found to be significant (lowest *p*-value 0.4). Due to the low number of samples, correlation between reaction forces and the TAI score during standing and transfer board wheelchair transfers were not calculated.

## 4 Discussion

Previous studies have identified the strong links between the performance of wheelchair transfers, risk of falling and the development of upper limb injuries [16, 17, 22].

Despite the fact that people with SCI represent only a small portion of wheelchair users [4], previous studies have mainly focused on this population when analysing the relationship between wheelchair transfers, falls and upper limb pain [5–8]. Additionally, although the use of transfer board has been recommended in order to reduce the load on the upper limbs during sitting wheelchair transfers its efficacy has never been tested [12]. To our knowledge, our study is the first to investigate the impact of sitting or standing technique and transfer board use on the overall transfer quality and the vertical reaction forces underneath both leading and trailing hands.

Results from our study confirm the expectation that standing and transfer board transfers will exhibit lower reaction forces under both hands when compared with sitting transfers. However, this difference was only significant between sitting and standing transfers. Transfer boards were only partially effective in reducing the weight born by the upper limbs. However, individuals who used them more regularly than our participants might be able to gain a greater benefit from their use When comparing our results to previous studies which measured reaction forces during sitting pivot transfers we found that our mean and peak values are lower than what is described by both [5, 28].

Reasons for this discrepancy can be explained. First, the difference in mean forces can be explained by the fact that both [5, 28] monitored reaction forces during the transfer itself only, while we included the preparation phase in order to capture the occurrence of scooting motions. This resulted in a considerably larger window of time, lowering the mean value of reaction force for both leading and trailing hand. Second, the peak reaction forces were higher in [5], who reported forces of 44.5 BW% under the trailing hand and up to 39.6 BW% under the leading hand; compared to 36.8 and 32.4 BW% measured during our study. However, subjects for both [5, 28] were individuals with SCI, while participants in our study had different medical conditions that might have allowed them to bear more weight on their legs, hence reducing the load underneath their hands.

Although the risk of developing upper limb injuries might be lower for individuals performing standing and transfer board transfers – resulting in the lower peak forces we observed – their transfer quality scored poorly, which would put them at higher risk of falling. Results from our study showed that transfers performed with a transfer board or a standing technique tended to receive lower TAI scores, and this difference was found to be significant for standing transfers. Although this could be partially due to the individual characteristics of the study's participants or to a lower accuracy of the TAI to assess standing and transfer board transfers, it potentially represents an important clinical indicator of the increased safety of sitting wheelchair transfers. This was further confirmed by the positive correlation found between TAI score and reaction forces across different techniques as sitting transfers had higher reaction forces compared with the other groups, but they were also judged to have been performed better.

When looking at the correlation between the forces generated during sitting transfer performance and the total score of the TAI Part 1, the overall trend of our findings confirms the results presented in [27]. However, the negative correlation was found to be non-significant. Reasons are likely related to the fact that both the set-up of the experiment and the number of TAI's items included in the analysis were different between the two studies. Additionally, the authors in [27] evaluated the TAI against kinetics variables such as specific joint reaction forces and moments rather than global reaction forces. The position of each joint and the presence of shear forces could easily be responsible of the discrepancy between the results.

Results presented in this study highlight some important differences between transfers performed with sitting, standing technique or with the aid of a transfer board and the effect that these differences might have on common risk factors associated with wheelchair transfers. Nonetheless, inherent limitations of the study suggest caution in the interpretation of the results, in particular the small sample size. Additionally, only one type of transfer was examined in this study and generalisation to different real-life situations such as transfers performed between the wheelchair and a car seat or a bathtub cannot be assumed. We would recommend a larger study which looks to categorise a range of transfer types with a larger population.

## 5 Conclusion

Transferring in and out of the wheelchair is an important activity for wheelchair users and has been previously associated with risk of falling and development of upper limb injuries. Previous studies have mainly focused on the relationship between these risk factors and the performance of sitting transfers by individuals with SCI. In this study, we extend the investigation to the relationship between reaction forces and transfer quality among individuals with different disabilities performing transfers with standing, sitting technique and using transfer boards. Although sitting transfers generated higher reaction forces which might lead to a greater risk to develop upper limb injuries they also seem to be of better quality, potentially resulting in a decreased risk of falling compared to other techniques.
